# Applied Biological and Physicochemical Activity of Isoquinoline Alkaloids: Oxoisoaporphine and Boldine

**DOI:** 10.3390/molecules170910958

**Published:** 2012-09-12

**Authors:** Eduardo Sobarzo-Sánchez, Patricio González Soto, Cristóbal Valdés Rivera, Georgina Sánchez, María Eliana Hidalgo

**Affiliations:** 1 Department of Pharmacy and Pharmaceutical Technology, Faculty of Pharmacy, University of Santiago de Compostela, Santiago de Compostela 15782, Spain; 2 Faculty of Sciences, University of Valparaíso, Valparaíso 33449, Chile; 3 Faculty of Pharmacy, University of Valparaíso, Valparaíso 33449, Chile

**Keywords:** antioxidant capacity, oxoisoaporphines, photoprotection, toxicity, singlet oxygen

## Abstract

The aim of this study was to determine the electronic influence of substituent groups and annelated rings such as oxazole-oxazinone on the physicochemical and photoprotection, antioxidant capacity, toxicity and singlet oxygen photosensitization biological properties of isoquinoline alkaloid frameworks. Thus, oxoisoaporphine derivatives **1**–**5** and 3-azaoxoisoaporphine (**6**), some of them with phenolic structures, did not present any antioxidant capacity, possibly either by formation of keto-enol tautomerism species or the formation of unstable free radicals. Due to the singlet oxygen quantum yields (Φ_Δ_) near to unity, and greater photostability than phenalenone, oxoisoaporphines **4**–**6** may be considered as photosensitizers for singlet oxygen production and can be used as new universal study tools. The biological application as antibacterial agents is an important and possible tool in the study of compounds with low cytotoxicity and high reactivity in antineoplastic chemotherapy. On the other hand, when boldine and its annelated derivatives **B1**–**4** are irradiated, a photoprotector effect is observed (SPF = 2.35), even after 30 minutes of irradiation. They also act as photoprotectors in cell fibroblast cultures. No hemolysis was detected for boldine hydrochloride and its salts without irradiation. In solutions irradiated before incubation (at concentrations over 200 ppm) photoproducts were toxic to the nauplii of *Artemia salina.*

## 1. Introduction

The isoquinoline alkaloids referred to as oxoisoaporphines (7*H*-dibenzo[*de,h*]quinolin-7-one) are isolated from *Menispermum dauricum* DC roots, which is the only known natural source of these alkaloids. This plant is known in China as Bei-Dou-Gen [1], and in Japan as Kohmori-Kazura [2]. The rhizomes of these plants are used in traditional Chinese medicine as analgesics and antipyretics for the treatment of sore throats, colitis, dysentery and rheumatic arthralgia [3], reasons why the plant is officially included in the China Pharmacopeia [4]. Studies and reports related to these compounds are scarce, but since the 1980s interest has been growing; it use in various pathological conditions has been studied and its antitumor activity is recognized [5] together with a beneficial activity in the cardiovascular system, acting as an antiarrhythmic-drug [6] and with dopaminergic activity in the D1 and D2 receptors in the central nervous system. Another study found that phenolic compounds decreased lipid peroxidation and enhanced the activity of SOD (superoxide dismutase) [7], as well as having anti-inflammatory action. This scaffold has a great potential with several pharmacological properties, however, its photochemical activity linked to the biological application as antineoplastic drugs has not been studied.

In this context, phenalenone (1*H*-phenalen-1-one) is used as a sensitizer in photochemistry and photobiology. This compound is soluble in a variety of solvents and the quantum yield, measured experimentally, is close to unity in the solvents used. According to these characteristics, it was proposed that phenalenone could be a universal reference singlet oxygen sensitizer [8]. In recent years, a new type of phytoalexins there have been isolated and characterized, whose phytoanticipyne structure is based on the skeleton of phenalenone. These are the 4- and 9-phenylphenalenone derivatives whose formation in response to fungal infection of banana plants has recently been described [9]. Compounds with a structure based on the phenalenone skeleton are normal metabolites in various plants and microorganisms [9,10]. These authors demonstrated that the presence of the natural phenalenone skeleton of these derivatives gave them singlet oxygen photosensitization capacity, and that its antifungal activity is increased significantly by the presence of light. Moreover, they found that phenylphenalenone derivatives having higher quantum yield developed higher antifungal activity under illumination, allowing them to establish a relationship between the ^1^O_2_ generation and the plant defence activity against a pathogen. Given the large number of potential pathogens that could provoke a variety of diseases, plants have developed defense strategies that can be classified as physical or biochemical. Thus, among biochemistry strategies there are several: the generation of reactive species derived from molecular oxygen, the superoxide ion [O^−^**^•^**], the hydrogen peroxide [H_2_O_2_], the hydroxyl radical [OH**•**] and the electronic state of molecular singlet oxygen [O_2_^1^] [11].

**Figure 1:**
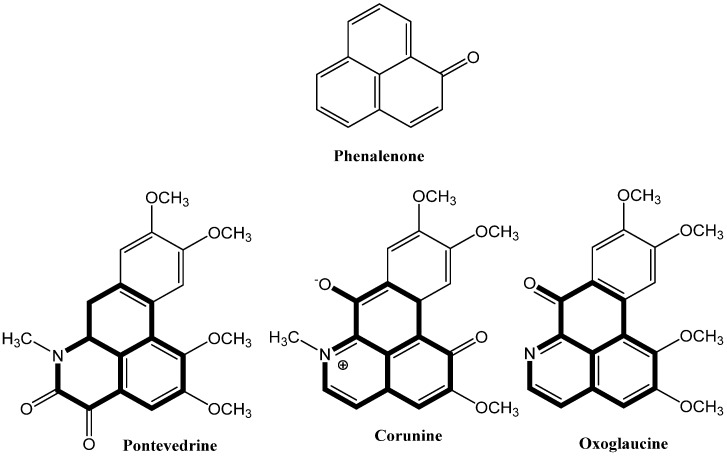
Chemical structures of phenalenone and oxoaporphines.

The singlet oxygen sensitization by the isoquinoline alkaloids derived from glaucine, namely oxoglaucine, corunine, pontevedrine has been studied [12,13] (Figure 1). Based on these reasons, it is necessary to study and provide natural compounds that are structurally related to phenalenone, that are efficient and able to sensitize singlet oxygen with photoquantum yields close to unity, greater photostability than phenalenone and possibly phototoxic to pathogens like the oxoaporphines [11]. Therefore, compounds such as oxoisoaporphine derivatives that include a phenalenone moiety, could be excellent candidates as possible singlet oxygen generators and for consideration either as effective antifungals or against other pathogens.

On the other hand, boldine [(*S*)-2,9-dihydroxy-1,10-dimethoxyaporphine], the major alkaloid present in the leaves and bark of the Chilean boldo tree (*Peumus boldus* Molina, Monimiaceae), has been characterized in the past few years as an antioxidant that effectively protects different systems against free-radical-induced lipid peroxidation and enzyme inactivation [14]. Boldine and other related aporphine alkaloids have been shown to behave as potent antioxidants in a number of experimental models. Pharmacological activities such as cyto-protective, anti-tumoral promoting, anti-inflammatory, antipyretic and antiplatelet activities have been associated with the ability of boldine to scavenge highly reactive free radicals [15].

In a previous paper [16], the photostability and photoprotective activity of boldine and glaucine were studied; both are photounstable under irradiation. However, the aporphine alkaloid structure remained unchanged and the photoproducts of glaucine exhibited a higher photoprotection factor (SPF) than the non-irradiated compounds. We also found [17] that boldine derivatives bearing substituent groups and annelated heterocycles with the aporphine framework have a greater photostability against UVB radiation. Thus, the compounds derived from boldine bearing oxazole and oxazinone annelated rings have promising photophysical and photochemical properties that could allow their possible use as sunscreens.

In this sense, there is evidence that oxoisoaporphines, some of which possess phenolic groups in their structures, inhibit lipid peroxidation; for this reason the antioxidant capacity of oxoisoaporphine derivatives with phenolic structure, might give us excellent *a priori* information about this property when not yet studied.

In this study, we evaluated the influence of substituent groups and annelated aromatic systems built on the boldine aporphine alkaloid framework: 8-nitroso-boldine hydrochloride (**B1**), 8-aminoboldine hydrochloride (**B2**), 9*H*-benzo[*de*]oxazoloboldine hydrochloride (**B3**), [1,4]oxazino[3',2':3,4]benzo-[1,2-g]benzo[de]boldine hydrochloride (**B4**), and several oxoisoaporphine derivatives and 3-azaoxoisoaporphine; 2,3-dihydro-5-methoxy-6-hydroxy-7*H*-dibenzo[*de,h*]quinolin-7-one (**1**), 5-methoxy-6-hydroxy-7*H*-dibenzo[*de,h*]quinolin-7-one (**2**), 5-hydroxy-7*H*-dibenzo[*de,h*]quinolin-7-one (**3**), 7*H*-dibenzo[*de,h*]quinolin-7-one (**4**), 5-methoxy-7*H*-dibenzo[*de,h*]quinolin-7-one (**5**), and 7*H*-benzo[*e*]perimidin-7-one (**6**) on several biological and physicochemical properties: photoprotection, antioxidant capacity, toxicity and singlet oxygen photosensitization, in order to explain the possibility of such isoquinoline compounds to act either as antimicrobial, antifungal or antineoplastic agents.

## 2. Results and Discussion

### 2.1. SPF *in Vitro* Test

Photoprotector capacity was calculated as Sun Protection Factor (SPF) with respect to homomethyl salicylate (SPF = 4). The SPF of the un-irradiated nitroso, amino, phenyloxazinone and oxazole boldine derivatives was similar to the SPF average value of un-irradiated boldine hydrochloride. After 30 min of irradiation the average value SPF of boldine hydrochloride and its derivatives was 2.35. No significant differences were observed attributable to the substituents (Table 1).

**Table 1:** Sun protection factor (SPF) *versus* irradiation time of boldine and its derivatives **B1**–**4**.

Irradiation time (min)	Boldine	B1	B2	B3	B4
0	6.30	6.33	6.51	6.39	6.43
5	4.12	4.08	4.20	4.00	4.02
10	3.71	3.93	4.07	3.95	3.97
15	3.70	3.90	4.00	3.80	3.86
20	3.23	3.25	2.68	2.71	2.77
25	2.96	2.80	2.58	2.68	2.70
30	2.33	2.35	2.41	2.31	2.36

The SPF of boldine hydrochloride and its derivatives in the salt form did not differ in any time range of the experiments, from time zero to 30 min of irradiation. Therefore boldine hydrochloride and its derivatives have a similar effect, expressed in the SPF value, as determined by the method of photoprotection *in vitro.* From zero to thirty minutes of irradiation, the SPF of boldine and its derivatives decreased from 6 to 2.35. In accordance with the Food and Drug Administration (FDA), sunscreens with SPF = 2 are acceptable as commercial products. Boldine and its derivatives, even after exposure to increasing irradiance doses, maintained their photoprotective properties.

### 2.2. Photohemolysis

The photoprotector effect of boldine hydrochloride was similar to that of the rest of its derivatives. Molecules containing nitroso groups were more toxic when irradiated before incubation due to the presence of the substituent group (NO), which may act by a free radical photodecomposition mechanism [18,19] (Table 2); however, the generation of photoproducts with a structure similar to nitrosoboldine hydrochloride (**B1**) is possible, which can be corroborated with the kinetics of photodecomposition in air and photo-consumption quantum yield [16].

**Table 2:** Hemolysis and photohemolysis (%) with preirradiated boldine hydrochlorideand its derivatives through red blood cell model.

Samples	% Hemolysis in darkness	% Hemolysis	% Photohemolysis
		preirradiated	
**Boldine**	0.00 ± 0.00	0.00 ± 0.00	2.31 ± 2.35 × 10^−3^
**B1**	0.00 ± 0.00	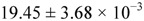	0.00 ± 0.00
**B2**	0.00 ± 0.00	0.00 ± 0.00	0.00 ± 0.00
**B3**	0.00 ± 0.00	0.00 ± 0.00	0.00 ± 0.00
**B4**	0.00 ± 0.00	0.00 ± 0.00	0.00 ± 0.00

Significant differences (*p* < 0.05, Mann-Whitney test) existed between the solutions of nitroso-boldine hydrochloride irradiated before incubation and those irradiated together with the red blood cell suspension.

### 2.3. Toxicity Test: Eggs of Artemia salina

The results (Table 3) indicate that LD_50_ average was 880 ppm for non-irradiated solutions, while for irradiated solutions the LD_50_ average was 620 ppm. UVB exerted a direct deleterious effect on nauplii (11% mortality) as was proven in experiments without oxoisoaporphines. Solutions irradiated before incubation produced the same effect, through photodecomposition by UVB radiation of boldine hydrochloride and derivatives in toxic photoproducts, on *Artemia salina.* Solutions irradiated before incubation produced a greater number of dead nauplii than irradiated solutions. The irradiated solutions therefore appear to exert a photoprotective effect up to a concentration of 200 ppm. At higher concentrations there was increased mortality, probably due to a high accumulation of toxic photoproducts.

**Table 3:** Median lethal dose (LD_50_, ppm) for boldine hydrochloride and its derivatives (**B1**–**4**) with the *Artemia salina* model.

Samples	Lethal dose 50 (LD_50_)	Lethal Dose 50 (LD_50_) Preirradiated	Lethal dose 50 (LD_50_) Irradiated
**Boldine**	900	400	685
**B1**	860	335	602
**B2**	900	305	580
**B3**	900	360	662
**B4**	850	366	560

### 2.4. Method of Fibroblast Cell Survival

The commercial formulations Bioderma Photoderm Spot ® Laboratoire Dermatologique (SPF 1) and Anthelios XL ® La Roche-Posay (SPF 2) that were used in this study produced fibroblast survival rates around 96%.

In our experiments, preparations containing boldine and its derivatives darkened when irradiated. Previous studies showed that the photoprotection observed *in vivo* for boldine hydrochloride could be attributable in part to physical effects on the skin of guinea pigs due to photoproducts that showed a dark coloration, providing additional protection against UV radiation [20].

**Figure 2:**
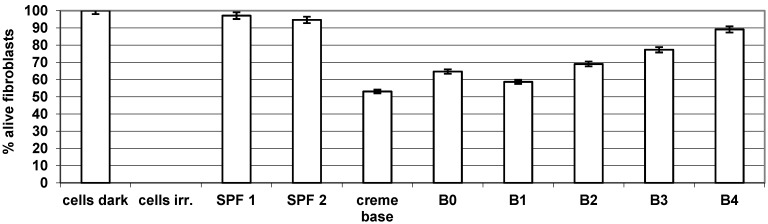
Photoprotector capacity (% alive fibroblasts) of SPFs commercial formulations compared with boldine derivatives **B1**–**4**.

The vehicle used to the study was the Novobase cream, which is made of a mixture of alcohols such as cetyl alcohol and stearyl alcohol and had a strong effect on cell survival, which was around 50%. Oxazole boldine hydrochloride (**B3**) and phenyloxazinone boldine hydrochloride (**B4**) had a higher photoprotector capacity than fibroblast cells, although not statistically significant; possibly due to the effect of substituent-fused heterocyclic containing stable functional groups with a higher resonance effect (Figure 2). Previous studies demonstrated that boldine with oxazine and phenyloxazinone moieties had greater stability than boldine against UVB radiation.

### 2.5. Antioxidant Capacity and Singlet Oxygen Formation

2,3-Dihydro-5-methoxy-6-hydroxy-7*H*-dibenzo[*de,h*]quinolin-7-one (**1**) and 5-methoxy-6-hydroxy-7*H*-dibenzo[*de,h*]quinolin-7-one (**2**) did not present antioxidant capacity at either high or low concentrations in the β-carotene assay method with Trolox as reference (Table 4). In this case there is a direct influence by the presence of a carbonyl group which generates an intramolecular hydrogen bond between two oxygen atoms, whose balance may result in the presence of keto-enol tautomeric species.

**Table 4:** Antioxidant capacities (AA) of oxoisoaporphines **1**–**3** evaluated from protection of β-carotene.

Compounds	Concentration (μM)	AA
**1**	100	0.35
**2**	100	0.40
**3**	100	0.35
**Trolox**	100	89.72

For 5-hydroxy-7*H*-dibenzo[*de,h*]quinolin-7-one (**3**), the stabilization of the phenyl radicals by the resonance at successive stages with the electron located in one of the radical resonance structures, positioned on the nitrogen atom, destabilized the molecule electronically, which does not contribute to the good stabilization of the radical formed, resulting in the observed decrease in antioxidant capacity.

Nevertheless, oxoisoaporphines **4**–**6** synthetically obtained in best yield for further studies by our group, showed a surprising effectiveness with a high degree of photosensitization in the production of singlet oxygen [O_2_ (^1^Δ_g_)]. The photostability that the tested compounds exhibit can be extended to different derivatives that contain a certain type of substituent that could modulate the photosensibility and stability in varied solvents of diverse polarity. Thus, the use of apolar solvents such as toluene, cyclohexane, studied among others, as well as of a polar solvent like methanol, showed that for oxoisoaporphine derivatives **4**–**6**, the quantum yield (Φ_Δ_) for formation of singlet oxygen with reference to phenalenone, is near to unity and with a clear ideal behavior (Table 5). In contrast to the phenalenone, the quantum yield of **4** is practically independent of the polarity of the solvent, which suggests that this compound might be used also as a universal reference.

**Table 5:** Singlet oxygen quantum yield (Φ_Δ_) of oxoisoaporphine derivatives (**4**–**6**) and phenalenone (**Phenal**) in different solvents and varied polarity.

Solvent	Polarity	Φ_4_	Φ_5_	Φ_6_	Φ_Phenal_
**Cyclohexane**	0.006	0.95			0.92
**Toluene**	0.099	1.00	0.95	1.00	0.95
**Tetrahydrofuran**	0.207	1.00			0.87
***N*,*N*'-dimethylacetamide**	0.377	1.00	0.93		0.87
**Acetonitrile**	0.460	0.98		1.00	0.98
**Propylene carbonate**	0.475	1.00			1.00
**Methanol**	0.762	1.00	0.97	0.93	0.98
**2,2,3.3-Tetrafluoropropanol**	0.886			1.00	1.00

## 3. Experimental

### 3.1. Synthesis of Boldine Derivatives

**Figure 3:**
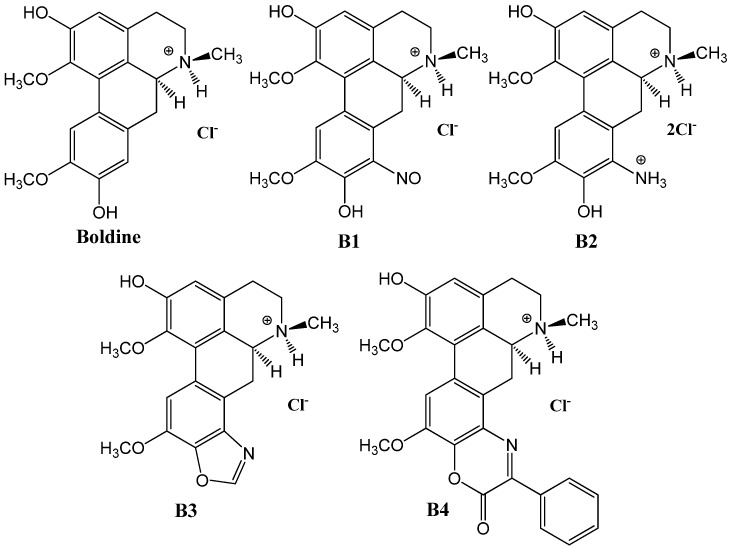
Chemical structures of boldine derivatives hydrochloride **B1**–**4**.

Boldine derivatives **B1**–**4** (Figure 3) were synthesized and characterized by standard methods [21,22]. Hydrochloride salts were prepared by dissolving the compounds in isopropanol and subsequently adding HCl, then precipitating the hydrochloride with ethyl ether. The reagents Brij 35 (Aldrich Laboratories, Milwaukee, WI, USA), dimethylsulfoxide, ethanol, methanol, tetrahydrofuran and acetonitrile (Merck, Santiago, Chile) were used. Distilled water adjusted to pH 3 and pH 10 with HCl and NaOH, respectively (Merck) was used.

### 3.2. SPF *in Vitro* Test

The photoprotector capacity in the UVB region was determined by measuring changes in light transmission of the solution of the compounds and homosalate (reference sunscreen, SPF = 4). The transmission spectra of the compounds (0.123 mg/5 mL) in a universal solvent, prepared by mixing 12.5 g methylene chloride, 37.5 g cyclohexane and 50 g isopropanol, were recorded in a spectrophotometer. The area under the curve was related to that of homosalate for SPF determination. A bank of six lamps, providing a mean irradiance of about 0.50 mW/cm^2^, was used as UVB radiation source. Irradiance measurements were made with a radiometer.

### 3.3. Method of Fibroblast Cell Survival [23]

Human fibroblast cells provided by the Laboratory of Physiological Chemistry and Immunology (Faculty of Pharmacy, University of Valparaíso) were grown in a flask seeded with culture medium [Dulbecco’s Modified Eagle medium (DMEM)/Ham’s F-12] supplemented with fetal calf serum (PAA), penicillin 10 U/L, streptomycin 100 µg/mL and amphotericin B 2.5 pg/mL (PAA). Of the ten plaques containing fibroblasts, one was left in darkness without irradiation and the remaining nine were irradiated with six lamps (UVB TLK 40W/12) with an irradiance of 0.50 mW/cm^2^ for 30 min, using approximately 40 minimal erythema dose. Nine of the six-well plates were irradiated, one without any protection, one completely and homogeneously covered on its upper face with a sun protector (SPF > 50, Bioderma ®), one completely and homogeneously covered on its upper face with a sun protector (SPF > 50, La Roche-Posay ®), one completely and homogeneously covered on its upper face with only a cream base (vehicle), one completely and homogeneously covered on its upper face with a cream base containing only boldine hydrochloride. Four of the six-well plates were completely and homogeneously covered on their upper faces with a cream base that contained only one derivative of boldine hydrochloride each. The absorbance was determined in a 96-well plates plate reader (Sensident Scan, Merck, Darmstadt, Germany) at 540 nm; the percentage of photoprotection of each boldine hydrochloride derivative was then calculated.

### 3.4. Photohemolysis

Red blood cells of healthy adult donors were used. Shortly after collection the heparinized blood was centrifuged at 2,000 *g* and the plasma and buffy coat were discarded. The remaining red cells were washed three times with an isotonic solution (0.15 M NaCl in 0.01 M sodium phosphate (PBS), pH 7.4). The red cells were resuspended to approximately 2% v/v, kept at 6 °C and used in the next 72 h. The percentage of hemolysis was determined immediately after irradiation and exposition to pre-irradiated solutions by measuring the hemoglobin liberated in the medium from solutions containing 0.4% red cells [24]. The measurements were carried out spectrophotometrically after centrifugation at 2,000 *g*, and the absorbance recorded at 540, 560, 577, 630 and 700 nm. The concentrations were evaluated according to the Winterbourn equation [18].

### 3.5. Toxicity Test: Eggs of Artemia saline

*Artemia salina* (Class: Crustacea, Subclass: Branchiopoda; Super order: Anostraca, Family: Artemidae, Genus: *Artemia*) cysts were incubated in filtered (micropore 0.22 μm) sea water and oxygenated for 45 min at 30 °C in a thermo regulated bath and adjusted to pH 8 with NaOH 0.1 M. After 24 h, the eclosd nauplii (first stage of *Artemia saline*) are in an appropriate condition for toxicity tests [25].

### 3.6. Synthesis of Oxozhines

**Figure 4:**
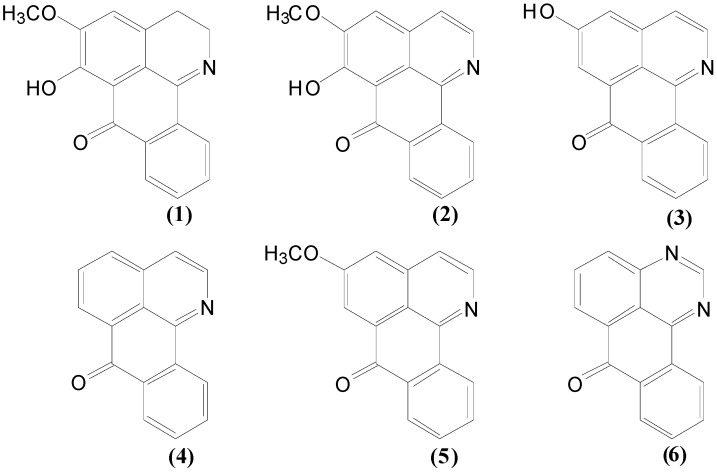
Chemical structures of oxoisoaporphine derivatives **1**–**6**.

Oxoisoaporphine derivatives **1**–**6** (Figure 4) were synthesized and characterized by standard methods [26].

### 3.7. Singlet Oxygen Production

The singlet oxygen production [O_2_ (^1^Δ_g_)] was assessed using a spectrophotometer system based on a life time of fluorescent 55 fluorescence PicoQuant Fluotime 200. Using an excitation laser Nd: YAG pumped by diode CryLas FTSS355-Q, emitting laser pulses at 355 nm, repetition rate of 10 kHz, pulse width of 1 ns and power 5 mW, J1J corresponding to 0.5 per pulse. The emission is collected at 90°, passed through a monochromator, and detected with a photomultiplier Hamamatsu H9170 near-IR-45. All photons are counted with a multichannel scaler PicoQuant’s Nanoharp 250. The histograms are analyzed using the software FluoFit, 60 also PicoQuant. 

### 3.8. Singlet Oxygen Quantum Yield

In order to determine the singlet oxygen quantum yield (Φ_Δ_) of the molecules under study, the technique of phosphorescence in the near IR (NIR) time-resolved has used. Values were obtained from time-resolved phosphorescence at 1,280 nm, which comes from the relaxation of singlet oxygen to its fundamental state. Phosphorescence signals are obtained and satisfy the equations describing the kinetics.

### 3.9. DPPH

The scavenger activities of various antioxidants were determined using the free radical 1,1-diphenyl-2-picrylhydrazyl (DPPH). In its radical form, DPPH has an absorption band at 515 nm which disappears upon reduction by an antioxidant compound [25,27].

### 3.10. Autooxidation of β-Carotene [28]

A diluted, oxygenated emulsion was prepared by the following procedure. A sample of crystalline β-carotene (2 mg) was dissolved in CHCl_3_ (10 mL) and an aliquot of this solution (1 mL) was added to purified linoleic acid (20 mg) and Tween 40 emulsifier (200 mg). After removal of CHCl_3_ in a rotary evaporator, oxygenated distilled H_2_O (50 mL) was added with vigorous stirring. An aliquot (5 mL) was pipetted into a spectrometer tube containing EtOH (0.2 mL) and the desired amount of antioxidant. The tubes were stoppered and placed in a H_2_O bath at 50 °C. Readings of absorptions at 470 nm were taken at regular intervals [29]. The antioxidant activity (AA) was evaluated from the equation:



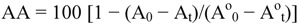



where A_0_ is the A measured at the begining of the incubation and A_t_ and A^o^_t_ are the A measured in the presence and absence, respectively, of the additive after incubation.

### 3.11. Statistical Analysis

The results of DPPH and DBC were analyzed by a nonparametric test (Kruskal-Wallis) with a confidence level of 95% using the program STATISTICA 7.0. For LC_50_ values probit analysis was used with the software MINITAB 15, with a confidence level of 95%.

## 4. Conclusions

Boldine and its derivatives in formulations exerted a photoprotective effect by acting as a screen, in a similar manner to their effect on irradiated guinea pig skin. In the red blood cell model, nitroso-boldine hydrochloride was the only compound that damaged the membrane, possibly by generation of more active radicals as degradation products. For boldine and derivatives a similar mechanism is possible, however the antioxidant defense systems would be capable of inhibiting erythrocyte damage.

In the *Artemia salina* model, at a concentration of 200 ppm, the oxoisoaporphine photodegradation products exerted a deleterious effect on nauplii. Oxoisoaporphines with phenol groups did not have antioxidant capacity due to the inactivation of the phenol group by hydrogen bond with the carbonyl group (compounds **1**,**2**). For **3** the effect of resonance stabilization produced an electronically unstable molecule, which possibly decreased the antioxidant capacity beyond the limit of the detection method. Nevertheless, due to the formation of singlet oxygen quantum yields (Φ_Δ_) near to unity, independent of the polarity of the solvent used in the studies, and greater photostability than phenalenone, these new types of isoquinoline alkaloids called oxoisoaporphines may be considered as new photosensitizers for singlet oxygen production and can be used as new universal references. Their application in antineoplastic therapy and as phototoxins against fungi is practicable and applicable to standard studies in this area.
